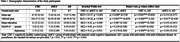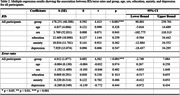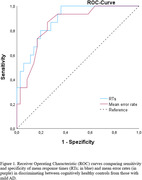# Emotional valence in rapid automatized naming for early detection of Alzheimer disease

**DOI:** 10.1002/alz.095442

**Published:** 2025-01-09

**Authors:** Sandra Lafenthaler, Yvonne Höller, Jürgen Bergmann, Margarita Kirschner, Vanessa Frey, Fabio Rossini, Eugen Trinka, Wolfgang Staffen

**Affiliations:** ^1^ Christian Doppler University Hospital, Paracelsus Medical University, Salzburg, Salzburg Austria; ^2^ Paris‐Lodron‐University of Salzburg, Salzburg, Salzburg Austria; ^3^ Center for Cognitive Neuroscience, Paris‐Lodron‐University of Salzburg, Salzburg, Salzburg Austria; ^4^ University of Akureyri, Akureyri, Akureyri Iceland; ^5^ Karl Landsteiner Institute for Neurorehabilitation and Space Neurology, Salzburg, Salzburg Austria; ^6^ Neuroscience Institute, Christian Doppler University Hospital, Paracelsus Medical University, Salzburg, Salzburg Austria

## Abstract

**Background:**

Alzheimer disease (AD) worsens naming abilities as the disease progresses. It is argued that traditional naming tests, commonly used to aid in staging AD severity, may overestimate semantic abilities. This study explored whether a more challenging naming task can distinguish between healthy adults and those with amnestic mild cognitive impairment (aMCI) or mild AD.

**Method:**

In a cross‐sectional study, 16 individuals with aMCI, 15 with mild AD, and 20 healthy controls (see Table 1 for demographic details) performed a rapid automatized naming (RAN) task with emotionally balanced object and action pictures. Naming performance was assessed by response times (RTs) and accuracy (error rates). Regression analyses examined the relationship between RTs/error rates and group, sex, age, education, anxiety, and depression. Receiver operating characteristic (ROC) curves evaluated the discriminative ability of the RAN task.

**Result:**

Patients with aMCI and mild AD showed significantly slower RTs and higher error rates compared to healthy controls (all p‐values < .001). Naming performance was especially impaired for negative stimuli compared to positive and neutral ones in healthy controls (p < 0.001) and aMCI patients (p < 0.01). However, this effect was absent in the AD group. Regression analyses revealed significant associations between RTs/error rates and group (both p‐values < 0.001), but not for age, sex, education, anxiety, and depression (see Table 2). ROC analysis demonstrated moderate diagnostic accuracy in classifying mild AD versus healthy controls (AUC of 0.887 for RTs; AUC of 0.854 for error rates; see Figure 1), but not in classifying aMCI versus healthy controls.

**Conclusion:**

The RAN task reliably detects variations in naming ability across different stages of cognitive decline associated with AD. We observed an inhibitory effect of negative valence on lexical retrieval processes, which appeared to be dependent on the degree of cognitive impairment, as it was no longer evident in our AD group. Further research with larger sample sizes is warranted. A significant limitation of our study is the lack of standardization of our visual stimuli. It is possible that the ratings for emotional valence derived from written words may not precisely reflect the emotional responses of our participants.